# Obstetrics; the Science and the Art

**Published:** 1867-05

**Authors:** 


Obstetrics; the Science and the Art. By Charles D. Meigs, M.D., lately Professor of Midwifery and Diseases of Women and Children, in Jefferson Medical College, of Philadelphia, etc., etc., etc. Fifth edition, revised, with one hundred and thirty illustratious. Philadelphia: Henry C. Lea. 1867.

This is a new and carefully revised edition of Dr. Meigs' well-known work on Midwifery. It is published in excellent style, with substantial binding, and contains 760 pages. The author has long been one of the ablest teachers and writers in our profession. And it is only necessary to remind our readers, that the work before us is everywhere recognized as a standard authority. For sale by W. B. Keen & Co., 148 Lake St.

